# Toxicity Evaluation of Sulfobetainized Branched Polyethyleneimine via Antibacterial and Biocompatibility Assays

**DOI:** 10.3390/toxics13020136

**Published:** 2025-02-14

**Authors:** Mehtap Sahiner, Selin S. Suner, Sahin Demirci, Ramesh S. Ayyala, Nurettin Sahiner

**Affiliations:** 1 Department of Bioengineering, Faculty of Engineering, Canakkale Onsekiz Mart University, Terzioglu Campus, 17100 Canakkale, Turkey; sahinerm78@gmail.com; 2 Department of Chemical, Biological, and Materials Engineering, University of South Florida, Tampa, FL 33620, USA; 3 Department of Chemistry, Faculty of Sciences, Canakkale Onsekiz Mart University, Terzioglu Campus, 17100 Canakkale, Turkey; sagbasselin@gmail.com; 4 Department of Food Engineering, Faculty of Engineering, Istanbul Aydin University, Florya Halit Aydin Campus, 34153 Istanbul, Turkey; sahindemirci@gmail.com; 5 Department of Ophthalmology, Morsani College of Medicine, University of South Florida, 12901 Bruce B. Downs Blvd, MDC21, Tampa, FL 33612, USA; 6 Department of Bioengineering, U.A. Whitaker College of Engineering, Florida Gulf Coast University, 10501 FGCU Boulevard South, Fort Myers, FL 33965, USA

**Keywords:** biocompatible polyethyleneimine, betainized PEI, zwitter ionic PEI, sulfobetainization, multiple betainization

## Abstract

Branched polyethyleneimine (PEI), possessing different types of amines—e.g., primary, secondary, and tertiary—in the polymer chains are well known for their antibacterial properties and DNA condensing ability, affording substantial advantages in many biomedical uses, including gene therapy. However, because of PEI’s toxicity, depending on the molecular weight, its widespread biomedical use is hindered. Therefore, in this study, PEIs with different molecular weights—i.e., 600, 1200, and 1800 g/mol—were modified with 1,3-propane sultone, undergoing a sulfobetainization reaction in a single step to attain a zwitterionic structure: sulfobetainized PEI (b-PEI). The sulfobetainization reaction was carried out twice to increase the zwitterionic repeating unit on PEI chains. The increasing number of SO_3_^−^ groups on the PEI chains was confirmed by the increased peak intensities around 1160 and 1035 cm^−1^ on the FT-IR spectrum, which are assigned to symmetric and asymmetric S=O peaks. The elemental analysis results for first- and second- betainization PEIs, abbreviated as b^1^-PEI and b^2^-PEI, respectively, were revealedthe increased wt% of S confirming the successful multiple-sulfobetainization of the PEI chains. Thermal stability analyses of PEIs and their corresponding multiple-sulfobetainized forms showed that multiple-sulfobetainization reactions increased the thermal stability of bare PEI chains. PEIs with lower molecular weights exhibited more antimicrobial properties. As PEI is sulfobetainated, its antimicrobial properties can be further adjusted via sulfobetainization (once or twice), or by adjusting the corresponding solution pH, or by protonating them with different acids with different counter anions. The cell toxicity of PEI on L929 fibroblast cells was slightly increased by increasing the molecular weight of the PEI, but all forms of sulfobetainized PEIs were found to be safe (no toxicity), even at 1000 µg/mL concentrations.

## 1. Introduction

Researchers have recently been focusing on zwitterionic polymers, including sulfobetainized polymers and particularly amine-based options, due to their potential in biomedical applications [1,2,3,4,5]. The unique zwitterionic structure of these materials contributes to their remarkable properties, including high biocompatibility, resistance to fouling, and robust stability, making them suitable candidates for various uses in the medical field [6,7,8,9]. The sulfobetainized polymers are promising vehicles for gene delivery due to their retained ability to establish electrostatic interactions with negatively charged nucleic acids, including DNA and RNA [10,11]. The zwitterionic properties of these polymers contribute to a decrease in toxicity and immune response when compared to traditional cationic polymers [12]. Furthermore, these materials are considered to be instrumental in stabilizing genetic material during systemic circulation and enhancing cellular uptake through endocytosis [13,14]. Recent studies have also focused on the utilization of pH-sensitive sulfobetainized polymers, which are engineered for the targeted release of genetic material, specifically in acidic microenvironments, which are characteristic of many tumors [15,16]. This innovative strategy aims to improve the efficacy of gene delivery systems by ensuring that therapeutic genes are released precisely at the sites of need, thereby maximizing their potential impact in cancer treatment.

Sulfobetainized polymers are also emerging as innovative materials for the development of controlled drug delivery systems [16,17,18]. Their intrinsic hydrophilicity and capacity to respond to various external stimuli, including pH, ionic strength, and temperature, enable the precise regulation of drug release [19,20,21,22]. For instance, hydrogels that encapsulate therapeutic agents and are composed of these polymers can release their contents in specific physiological conditions, thereby enhancing therapeutic efficacy while minimizing adverse effects [23,24]. Additionally, the utilization of sulfobetainized nanoparticle coatings effectively inhibits protein adsorption, which is essential for prolonging the circulation time of these nanoparticles within the bloodstream [25,26,27,28]. This characteristic improves the pharmacokinetics of the drug delivery system and facilitates targeted delivery to specific tissues, thereby enhancing the overall therapeutic outcome. Consequently, the distinctive properties of sulfobetaine polymers make them valuable assets in the advancement of drug delivery technologies.

In the area of tissue engineering, these polymers function as biocompatible scaffolds that promote cell adhesion, proliferation, and differentiation [3,29,30,31]. Their tunable mechanical and chemical properties facilitate the development of three-dimensional scaffolds that closely mimic the extracellular matrix [32,33]. This innovative application presents considerable potential for the regeneration of various tissues, including skin, cartilage, and cardiac tissues [30,33,34,35]. The incorporation of these polymers in tissue engineering is crucial as they create a conducive environment for cellular processes that are vital for tissue regeneration [36,37]. By emulating the extracellular matrix through their customizable attributes, these scaffolds can effectively enhance the healing and restoration of diverse tissue types, thereby advancing therapeutic approaches in regenerative medicine.

Moreover, sulfobetainized polymers are increasingly utilized in developing antimicrobial coatings for various medical devices, including catheters, surgical instruments, and implants [38,39,40,41,42]. The zwitterionic characteristics of these surfaces effectively inhibit bacterial adhesion and the subsequent formation of biofilms, thereby significantly reducing the risk of infections associated with these devices [38,40,41]. Certain formulations may also incorporate antimicrobial agents that are released in a controlled manner, providing an additional layer of protection against potential infections. Furthermore, the integration of sulfobetainized polymers into biosensors distinctly enhances their sensitivity and accuracy by minimizing nonspecific protein adsorption [25,43,44,45,46]. This property is particularly beneficial for diagnostic devices, especially when analyzing complex biological fluids such as blood or plasma, where contaminants can negatively impact sensor performance. Overall, the distinctive properties of sulfobetainized polymers not only improve the functionality of medical devices by mitigating infection risks but also enhance the reliability of biosensors in challenging environments. Their capacity to maintain functionality in the presence of biological materials underscores their importance in advancing medical technology and improving patient outcomes.

The antibacterial, antifungal, and antiviral activity of betainized PEI60K were reported by our group in an earlier study [12]. Differing from the earlier report [12], here, the branched PEIa with varying molecular weights (MWs)—e.g., 600, 1200, and 1800 g/mol—were used to prepare their corresponding zwitterionic forms via a sulfobetainized reaction involving a single-step simple modification reaction using 1,3-propane sultone as a modifying agent at ambient temperature. Moreover, the sulfobetainization of amine groups on PEI chains was conducted twice to increase the number of the zwitterionic units on PEI chains. Furthermore, the cytotoxicity of all PEIs with different MWs and their corresponding first- and second-sulfobetainization forms on L929 fibroblast cells were tested via an MTT assay. Additionally, the changes in the antibacterial activity of PEIs with different MWs upon multiple sulfobetainization reactions were examined against Gram-negative *Escherichia coli*, Gram-positive *Staphylococcus aureus*, and yeast *Candida albicans* via micro-dilution tests.

## 2. Materials and Methods

### 2.1. Materials

Polyethyleneimine (PEI, i.e., polyethyleneimine solution average M_n_ ~600, 1200, and 1800 by LS, 99%, Aldrich, Milwaukee, WI, USA) was used as received. The modifying agent, 1,3-propane sultone (PS, 98%, TCI, Montgomeryville, PA, USA) was used to prepare the sulfobetainized PEI (b-PEI) chains. Distilled water (GFL 2108) was used to prepare all aqueous solutions. High-purity acetone (>99, Thermo Fisher, Waltham, MA, USA) precipitated the b-PEI chains. Boric acid (BA, ≥99.5%, Crystalline/Certified ACS, Fisher Chemical™, Waltham, MA, USA), citric acid monohydrate (CA, (Granular/Certified ACS), Fisher Chemical™), and hydrochloric acid (37 wt%, ACS reagent, Sigma-Aldrich, St. Louis, MO, USA) were used for protonation process.

L929 fibroblast cells (Mouse C3, a connective tissue) were obtained from the SAP Institute (Ankara, Turkey). In the cell culture, Dulbecco’s modified eagle medium (DMEM, with 4.5 g/L glucose, 3.7 g/L sodium pyruvate, L-Glutamine 0.5 g/mL), fetal bovine serum (FBS, heat-inactivated), and penicillin/streptomycin (10,000 U/mL penicillin, 10 mg/mL streptomycin) were purchased from Pan Biontech (GmbH, Aidenbach, Germany). Trypsin (0.25%, EDTA 0.02% in PBS, Pan Biontech GmbH, Aidenbach, Germany) was used as received. As an MTT agent, 3-(4,5-dimethylthiazol-2-yl)-2,5-diphenyltetrazolium bromide was purchased from BioFroxx, Einhausen, Germany. *Candida albicans* (ATCC 10231), *Staphylococcus aureus* (ATCC 6538), and *Escherichia coli* (ATCC 8739) were used for antimicrobial studies. Potato dextrose agar (PDA, Becton, Dickinson and Company, Sparks, MD, USA) and potato dextrose broth (PDB, bioWorld, GeneLinx International, Inc., Dublin, OH, USA), as growth media for fungus, were purchased and used as received. Nutrient agar (NA) and nutrient broth (NB), as growth media for bacteria, were purchased from BD DifcoTM (Becton, Dickinson and Company, Sparks, MD, USA) and used as received.

### 2.2. Preparation and Protonation of Sulfobetainized PEI Chains

The sulfobetainization of PEI was conducted with minor modifications to the established methodology reported by our group [47]. In short, 5 g of PEI was dissolved in 45 mL of distilled water, followed by the addition of 4.1 g of PS in a 1:1 molar ratio to the primary amine groups in the PEI structure. Previous studies have indicated that the ratio of primary, secondary, and tertiary amines in branched PEI is 1:2:1 [48]. The resulting mixture was stirred at 500 rpm under ambient conditions for 12 h. Subsequently, the mixture was gradually introduced into approximately 2 L of acetone, facilitating the precipitation of sulfobetainized PEI over 12 h. The supernatant was then discarded, and the remaining precipitate was dried in an oven at 50 °C for two days. This final product was designated b^1^-PEI, which was consistently employed throughout this study.

In the process of double sulfobetainization, 5 g of b^1^-PEI was placed into a 100 mL round-bottom flask and subsequently dissolved in 50 mL of distilled water. Following this, 4.1 g of PS was introduced into the resulting solution. The same parameters and conditions—i.e., the reaction time and the precipitation and drying conditions—were conducted as previously described for b^1^-PEI. The resultant product was designated as b^2^-PEI, and this designation was consistently employed throughout this research.

The b-PEI chains treated distinct protonation processes through the treatment of citric acid (CA), boric acid (BA), and hydrochloric acid (HCl). Specifically, solutions containing 40 wt% b-PEI chains were prepared in 10 mL of 0.1 M CA, 0.1 M BA, and 0.01 M HCl aqueous solutions, respectively. Following a stirring period of six hours at ambient temperature, the resultant solutions were gradually introduced into 500 mL of acetone while maintaining a stirring speed of 500 rpm. Ultimately, the synthesized b-PEI-CA, b-PEI-BA, and b-PEI-HCl solids were dissolved in biological saline solution (BSS) at a concentration of 40%, thereby rendering them suitable for subsequent applications.

### 2.3. Characterization of b-PEI Before and After Modification

To confirm the sulfobetainization reaction of polyethyleneimine (PEI), Fourier transform infrared radiation (FT-IR, Nicolet iS10 model from Thermo Fisher Scientific, Waltham, MA, USA) spectroscopy was utilized. FT-IR spectra were collected from the samples before and after the betainization (modification) reactions. For each sample, spectra were recorded using a total of 16 scans, with a resolution of 4 cm^−1^, spanning a frequency range from 650 to 4000 cm^−1^.

The thermal degradation of PEI before and after modification was investigated using a Thermogravimetric Analyzer (TGA, SII TG/DTA 6300, Tokyo, Japan). The analysis was conducted in a nitrogen atmosphere at a flow rate of 20 mL/min, with a heating rate of 10 °C/min, over a temperature range of 100 to 600 °C.

The elemental composition of bare and sulfobetainized PEI chains was also determined via an elemental analyzer (Leco, CHNS-932, St. Joseph, MI, USA)

The zeta potential values of the PEI at different molecular weights (600, 1200, and 1800), and of b^1^-PEI and b^2^-PEI at different PEI molecular weights at different pH values at a concentration of 1 mg/mL, were determined using a zeta potential analyzer (Zetapals, 90 plus, Brookhaven Instrument Corp., Holtsville, NY, USA).

### 2.4. Antibacterial Properties of PEI-Based Structures

The antimicrobial activity of PEI (MW: 600, 1200, and 1800), b^1^-PEI, and b^2^-PEI at different MWs was determined by a microdilution test in broth against two bacterial strains and *Candida albicans*, in accordance with the literature [1]. Briefly, PEI, b^1^ PEI, and b^2^ PEI were dissolved in a BSS solution with an initial concentration of 40% by weight. Sample solutions with concentrations between 20–0.25% were added to 96 wells in nutrient broth medium and incubated for 24 h. At the end of incubation, the minimum inhibitory concentration (MIC) was determined. According to the literature, the minimum bactericidal concentration (MBC) of microorganisms in nutrient agar and potato agar was determined [1]. The sample solution incubated with microorganisms was shown on nutrient agar and potato agar in the Petri dishes, and the concentration, which showed no growth after 24 h, was determined as MBC. Amphotericin B, an antifungal agent, and ciprofloxacin, an antibacterial drug, were used as positive controls.

### 2.5. Cytotoxicity of PEI, b^1^-PEI, and b^2^-PEI

The cytotoxicity of branched PEIs, as well as of their b^1^-PEI and b^2^-PEI forms, were determined using an MTT assay on L929 fibroblast cells. The fibroblasts were cultured in DMEM supplemented with 10% (*v*/*v*) FBS and 1% antibiotics and seeded at 5 × 10^3^ cells per well in a 96-well plate. After 24 h incubation at 37 °C in 5% CO_2_, the adhered cells were treated with 100 µL of PEI-based solution in DMEM at 10, 50, 100, and 1000 µg/mL concentrations. As a control, 100 µL of fresh culture medium was used. The culture continued for 24 h more at 37 °C in 5% CO_2_. At the end of the incubation, the PEI solution was removed, and 100 µL MTT solution at 0.5 mg/mL concentration in DMEM interacted with the cells in the dark. After 4 h, the MTT solution was removed, and 200 µL of DMSO was added. Cell viability % was determined by the absorbance of the wells at 570 nm by a plate reader (Thermo, Multiskan Sky, Waltham, MA, USA). The analysis was applied three times and given with standard deviations. Ordinary one-way ANOVA and Dunnett’s multiple comparison tests were performed via GraphPad Prism 10 software to test statistical significance. The difference was considered significant for *p*-values of * *p* < 0.05, ** *p* < 0.01, *** *p* < 0.001, and **** *p* < 0.0001 vs. control.

## 3. Results and Discussion

### 3.1. The Synthesis and Structural Characterization of Betainized b-PEI Chains

**Figure 1:**
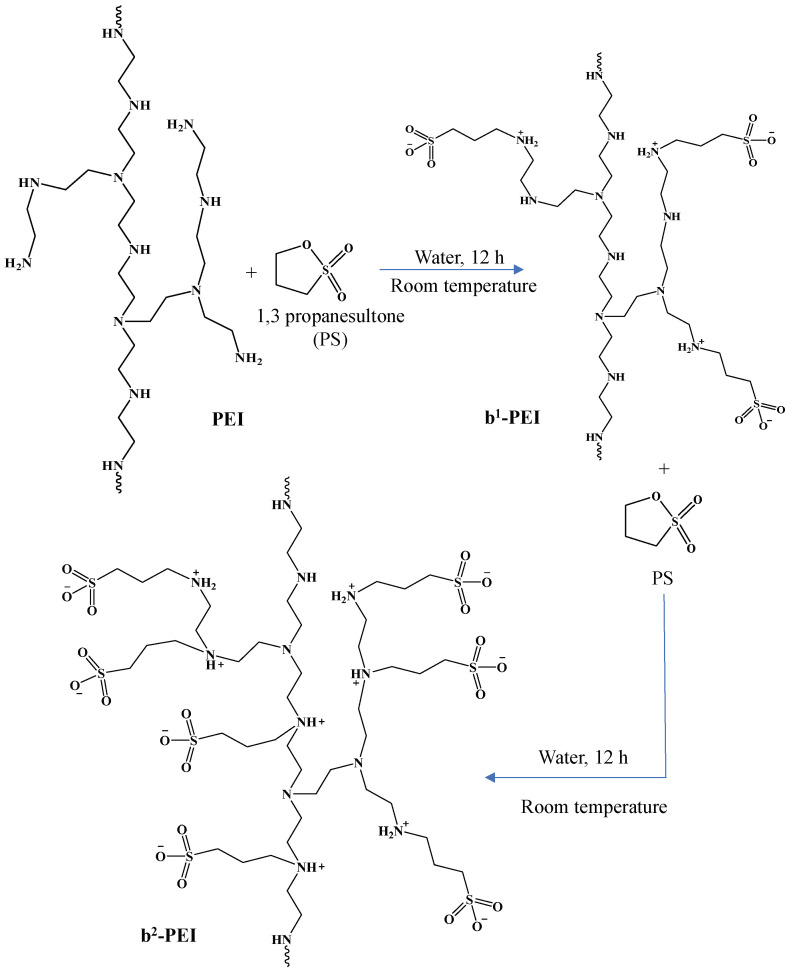
Schematic presentation of the double betainization of b-PEI chains.

The multiple sulfobetainization reaction of PEI is illustrated in Figure 1. The PEI chains containing primary, secondary, and tertiary amines in their structure provided an excellent opportunity to prepare sulfobetainized polymers. In this context, the lone pair of electrons from the nitrogen atoms in the amine groups of PEI participates in a nucleophilic attack on the cyclic sulfonate ester, denoted as PS [49,50]. This interaction promotes the cleavage of the five-membered sulfonate ring, resulting in the formation of a protonated nitrogen atom and negatively charged sulfonate groups on the PEI chain [51,52]. The result of this reaction is the formation of sulfobetainized PEI, a zwitterionic structure characterized by the presence of both positively charged nitrogen and negatively charged sulfonate groups [49,52]. This transformation not only modifies PEI’s chains, rendering new properties; it also increases its potential applications across various domains by reducing the toxicity of bare PEI and increasing its biocompatibility. As mentioned, these kinds of betainized materials can be used in many biomedical fields, including tissue engineering and drug delivery systems.

Here, b^1^-PEI was prepared through the reaction of PS with primary amine groups on the PEI chains, which are more reactive than secondary and tertiary amines. Subsequently, the second betainization reaction was carried out to betainize more numbers ofprimary amines—as well as the secondary and tertiary amine groups—on PEI chains to obtain b^2^-PEI structures.

**Figure 2:**
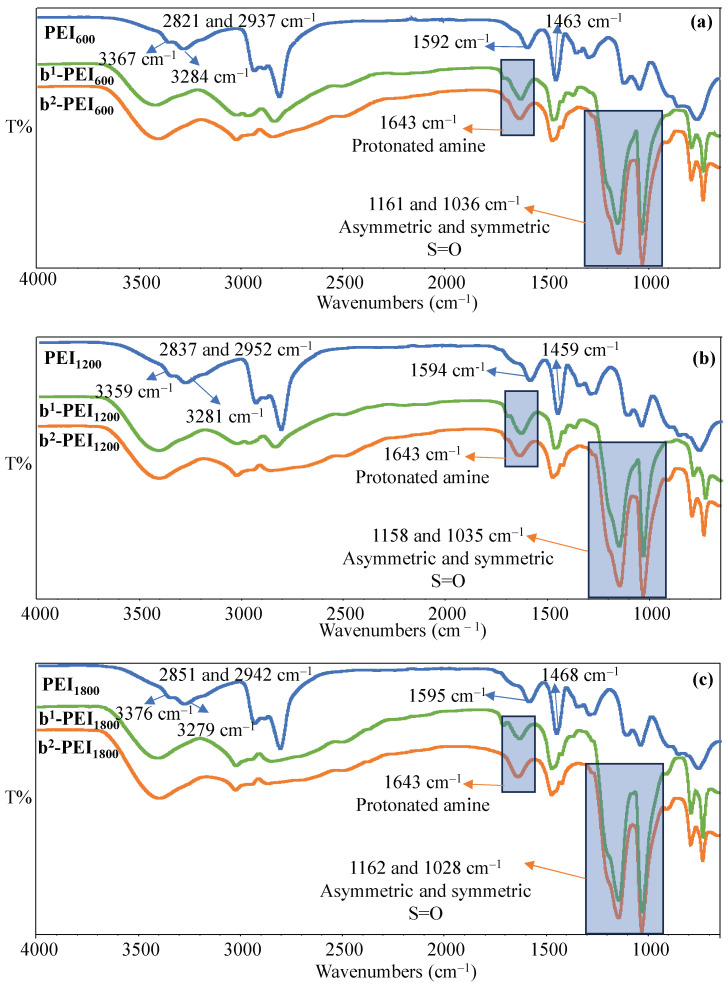
The FT-IR spectra of the prepared PEI, b^1^-PEI, and b^2^-PEI chains: (**a**) M_n_ = 600; (**b**) M_n_ = 1200; and (**c**) M_n_ = 1800 g/mol.

To confirm the betainization of branch PEI, the FT-IR spectra of PEI, b^1^-PEI, and b^2^-PEI were compared and are presented in Figure 2a–c. As seen in Figure 2, the FT-IR spectra of PEI_600_, PEI_1200_, and PEI_1800_ chains exhibited the following stretching vibrations: N-H bending for NH_2_ and NH vibrations around 1590 and 1450 cm^−1^; C-H stretching peaks for CH_2_ at 2800 and 2900 cm^−1^; and symmetric and asymmetric N-H stretching of NH_2_ at 3250 and 3360 cm^−1^, respectively.

On the other hand, upon betainization of PEI chains, new peaks were observed. In the FT-IR spectrum of both the b^1^-PEI and b^2^-PEI chains for all molecular weights of the PEI chains, the peaks at around 1160 and 1030 cm^−1^ were assigned to asymmetric and symmetric S=O peaks, respectively [47]. On the other hand, the peaks observed at about 1650 cm^−1^ are attributed to protonated amine groups after the betainization reaction of PEI chains [12]. The increased peak intensities at wave numbers of 1160 and 1030 cm^−1^ following the second betainization reaction is apparent; however, a definitive quantitative analysis continues to be elusive. While the increase in intensity at the specified stretching frequencies is evident following the second betainization reaction, it is not possible to determine a precise quantity at this stage. Therefore, the elemental analysis of all PEI, b^1^-PEI, and b^2^-PEI chains from various molecular weights of PEI, such as 600, 1200, and 1800 g/mol, were carried out, and the elemental composition results are summarized in Table 1. Moreover, the theoretically calculated wt% of C, H, N, O, and S are also given in Table S1. If a sulfobetainization reaction was accomplished 100%, the theoretical wt% of the S values would be expected to be 10.8% for the initial sulfobetainization and 15.4% for the subsequent betainization across all the polyethyleneimine (PEI) molecules of all molecular weights. However, as shown in Table 1, the wt% of the S of PEIs with different MWs is reduced as the MW is increased for first and second sulfobetainization reactions.

However, it was seen that the wt% of C, H, and N for PEI_600_, PEI_1200_, and PEI_1800_ have almost-similar ratios of C (around 55.5%), H (around 11.6%), and N (around 32.8%), respectively. On the other hand, after the first and second betainization reactions, the wt% of C, H, and N decreased, as expected, and the wt% of the O and S values increased upon betainization reaction due to the modification reaction of PS with the amine groups on the PEI chains. The wt% of S was determined to be 8.1% for b^1^-PEI_600_ and increased to 12.3% after the preparation of b^2^-PEI_600_. The wt% values of S for b^1^-PEI_1200_ and b^2^-PEI_1200_ were also determined as 6.8%, and 10.4%, respectively. Also, the determined wt% of the S values for b^1^-PEI_1800_ increased from 5.2% to 7.6% after the second betainization reaction for b^2^-PEI_1800_. The sulfobetainization degrees for PEI chains of different molecular weights were determined via a comparison of the theoretically calculated and experimentally determined wt% values of the S content of sulfobetainized PEIs. Accordingly, the wt% of the S values, which denote the degree of sulfobetainization for PEI_600_, was determined to be 74.3 and 79.9% at the end of the first and second sulfobetainization reactions, respectively. On the other hand, sulfobetainization degrees of 62.4 and 67.4% for PEI_1200_ and 47.7 and 49.3% for b1-PEI_1800_ and b^2^-PEI_1800_ were calculated, respectively. These observed decreases in the wt% of the S value based on the molecular weights of used PEI chains are associated with an increase in the molecular weight of the PEI chains employed. This suggests that the sulfobetainization reactions yield is higher for PEI chains of lower MW than for those with higher MWs. The observed trend indicates that the S wt% decreases with the increasing molecular weight of PEI; specifically, approximately 80% of the amine groups in PEI_600_, which corresponds to roughly 14 amines, are sulfobetainized at the end of two sulfobetainization reactions. In the case of PEI_1200_, about 70% of the amines, or about 21 amines, are sulfobetainized. For PEI_1800_, approximately 50% of the amines, totaling about 23, are sulfobetainized. These data suggest that the number of sulfobetainized amines within the polymer structure increases as the molecular weight of the PEI increases. Consequently, the wt% of the S values corresponding to the repeating units are ranked in the following order: PEI_1800_, PEI_1200_, and PEI_600_. This hierarchy emphasizes the correlation between MW and the efficiency of the betainization process, thereby highlighting the substantial influence that the selection of PEI molecular weight exerts on the resultant S% values.

**Figure 3:**
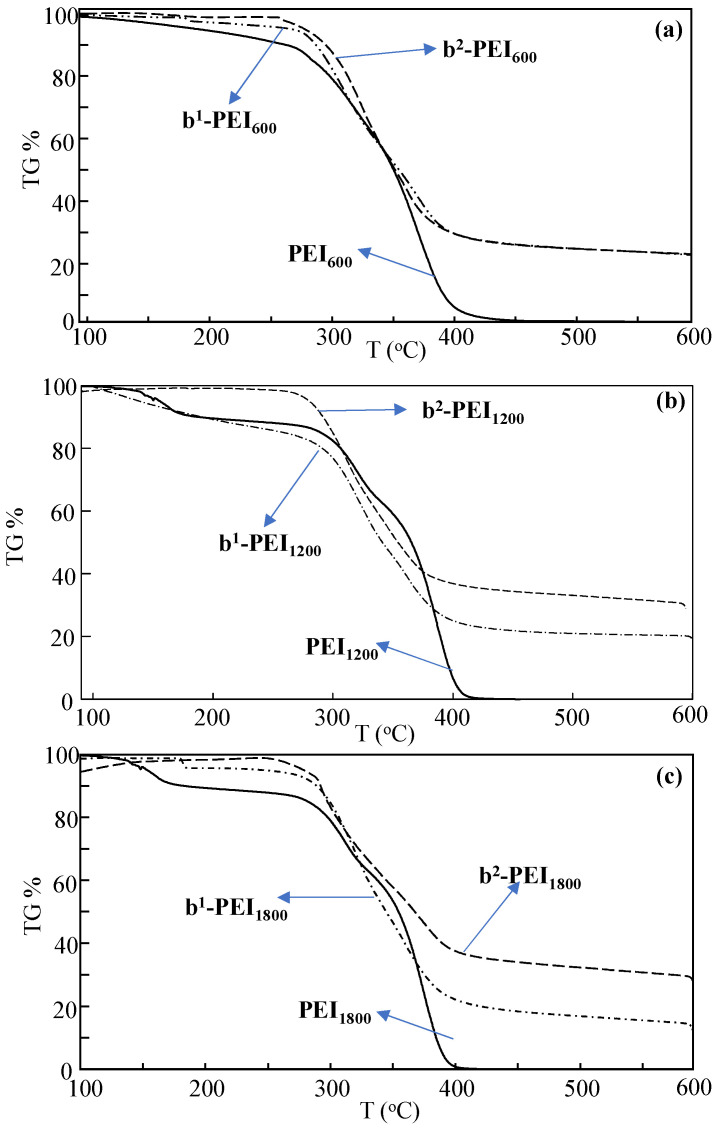
The TGA thermograms of PEI, b^1^-PEI, and b^2^-PEI chains prepared from (**a**) M_n_ = 600, (**b**) M_n_ = 1200, and (**c**) M_n_ = 1800 g/mol, respectively.

The thermal stabilities of the PEI and b-PEI prepared with different MWs of PEI chains were also compared and are shown in Figure 3. In Figure 3a, it is evident that PEI_600_ begins to thermally degrade at the temperature range of 100–250 °C, resulting in a weight loss of approximately 10%. A significant degradation phase occurred in the 260–400 °C range, and finally, there was a total weight loss exceeding 99% following heating from 500 °C to 600 °C. In contrast, b^1^-PEI_600_ exhibits a weight reduction of nearly 5% up to 270 °C, with degradation commencing between 275 °C and 400 °C, with a 70% weight decrease and a cumulative weight loss of approximately 77.2% at 600 °C. Conversely, b^2^-PEI_600_ remains stable until 250 °C, and a weight loss of 69.2% was observed in the 260–400 °C range.

The thermal stability of PEI_1200_, b^1^-PEI_1200_, and b^2^-PEI_1200_ were evaluated, and the results are illustrated in Figure 3b. In comparison to PEI_600_, PEI_1200_ demonstrated an initial thermal degradation in the temperature range of 120–180 °C, leading to a weight loss of 9.2%. This was followed by a significant thermal degradation occurring between 280 and 410 °C, resulting in a cumulative weight loss exceeding 99%. In contrast, b^1^-PEI_1200_ exhibited a weight loss of 17.5% within the temperature range of 100–280 °C, followed by a marked degradation phase from 285 to 435 °C, which accounted for a cumulative weight loss of 78.1%, and then an approximately 80% weight loss was recorded at 600 °C. Similarly, b^2^-PEI_1200_ did not display any degradation up to 260 °C; however, it exhibited a thermal degradation in the 265–410 °C range, resulting in a weight loss of 62.9%. The degradation continued up to 600 °C, ultimately leading to a total weight loss of 70%. The comparison of the thermal stability of PEI_1800_, b^1^-PEI_1800_, and b^2^-PEI_1800_ (Figure 3c) also showed almost similar tendencies with the PEI_1200_-based examples. It can be conclusively stated that the various sulfobetainization reactions applied to PEI chains of differing molecular weights have enhanced the thermal stability of the unmodified PEI chains, resulting in increasing temperatures at which thermal degradation initiates. The thermal degradation of sulfobetainized PEI in the 280–440 °C range could be due to the desulfonation of the molecules [53,54].

**Figure 4:**
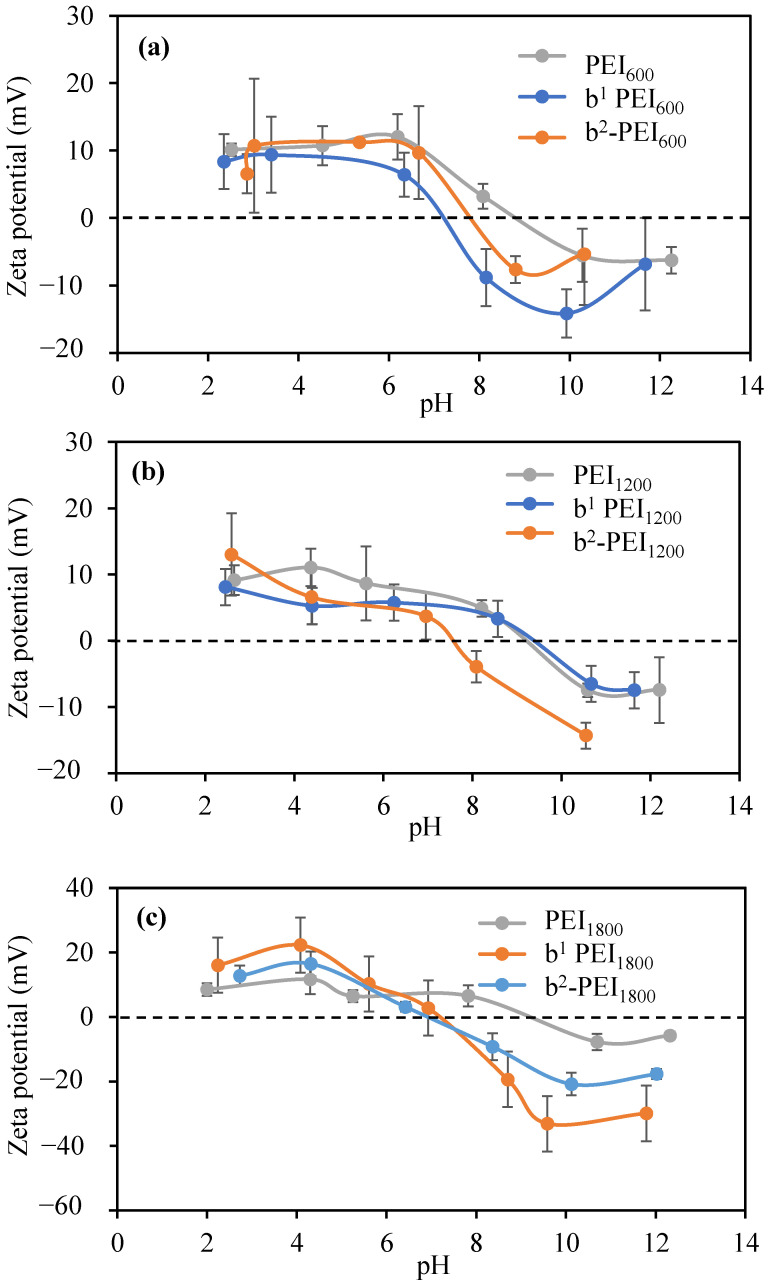
(**a**) Zeta potential measurements of PEI_600_, b^1^PEI_600_, and b^2^ PEI_600_ at different pHs in 0.01 M KNO_3_ solution; (**b**) zeta potential measurements of PEI_1200_, b^1^-PEI_1200_, and b^2^ PEI_1200_ at different pHs in 0.01 M KNO_3_ solution; and (**c**) zeta potential measurements of PEI_1800_, ^1^b-PEI_1800_, and ^2^b-PEI_1800_ in different pHs in 0.01 M KNO_3_ solution.

Figure 4a presents the zeta potentials of PEI_600_, ^1^b-PEI_600_, and ^2^b-PEI_600_ against pH. Upon the sulfobetainization reaction, the amine groups that are not protonated become protonated, and the second sulfobetainization ensures greater protonation of unprotonated amines while also introducing negatively charged sulphate groups. Therefore, in an acidic medium, the zeta potential for b-PEIs is significantly changed

The zeta potential of PEI_600_ is positive at low values down to pH 10, while it becomes negative after pH 10. As the zeta potential of ^1^b-PEI_600_ is calculated against pH, its IEP is calculated as 7.2. The IEP of ^2^b-PEI_600_ is determined as 7.5. Figure 4b gives the zeta potential values of PEI_1200_, ^1^b-PEI_1200_, and ^2^b-PEI_1200_ against different pH solutions. The zeta potential of PEI_1200_ at 1 mM KNO_3_ solution is determined to be −7.5 ± 1.0 mV, the pH of the solution was 10.6, and the IEP was determined to be 9.2. It can be seen that the sulfobetainization of PEI shifted the IEP to lower values. When the zeta potential of ^1^b-PEI_1200_ is graphed against pH, the isoelectric point (IEP) is calculated as 9.4. The IEP of ^2^b-PEI_1200_ is determined to be 7.6.

Figure 4c shows the zeta potentials of PEI_1800_, b^1^-PEI_1800_, and b^2^-PEI_1800_ prepared with PEI_1800_ against different pHs. To determine the IEP of b^1^PEI_1800_ and b^2^-PEI_1800_, the zeta potentials values of b^1^PEI_1800_, and b^2^-PEI_1800_ at different pH solutions were measured, and the results are shown in Figure 4c. The IEPs for b^1^-PEI_1800_ were measured at pH 7.1, and those for ^2^b-PEI_1800_ were measured at 6.9, respectively, indicating that upon betainization, PEIs are at roughly physiological pHs (7.4). The IEP of PEI_1800_ was determined at pH 9.2. It can be seen in Figure 4c that with the betainization process, the IEP decreases, and the polymer solution becomes more acidic.

Table 2 gives the zeta potentials of PEI, b^1^-PEI, and b^2^-PEI. Also, the pH of PEI, b^1^-PEI, and ^2^b-PEI was measured in a 1 mM KNO_3_ solution at a concentration of 1 mg/mL. According to the results, all the PEIs, with different MWs within 1 mM KNO_3_ at 1 mg/mL, have a solution pH of about 10.5, which is very similar to what is reported in the literature [55]. While the pH value of ^1^b-PEIs varies between 5.6 and 6.3, ^2^b-PEI varies between 2.6 and 3.0. Sulfonated twice, the pH is around 3, and as the betainization process lowers the corresponding solution pH, it slightly increases the zeta potential.

### 3.2. Antibacterial Properties of Sulfobetainized PEI

Disinfectants are widely believed to be effective in preventing and suppressing infectious diseases by blocking the pathogenic microorganisms’ direct path to humans. Therefore, coating delicate items with antibacterial coatings could successfully stop microorganisms from growing on their surfaces and stop harmful microorganisms from indirectly coming into contact with humans [56].

Before- and after-betainiezation PEIs with different MWs, they were tested for their antibacterial potency against common pathogens such as *Escherichia coli* (ATCC 8739), *Staphylococcus aureus* (ATCC 6538), and *Candida albicans* (ATCC 10231), and the results are summarized in Table 3 and Table 4, respectively. As can be seen in Table 3, as the molecular weight of PEI increases, its antimicrobial activity decreases. For example, for *C. albicans*, the MIC value of PEI_600_ was found to be 0.5 mg/mL; for PEI_1200_ and PEI_1800_, the MIC values were found to be 1 mg/mL. PEIs with lower MW possess higher antibacterial potency than higher-MW PEIs; e.g., an MIC value of 5 mg/mL against *C. albicans* for PEI_60000_ was reported [12].

Amphotericin B (against fungi) and ciprofloxacin (against bacteria) were used as positive controls. MIC and MBC values of 0.025 mg/mL against *C. albicans* and an MIC value 0.00625 mg/mL and an MBC value of 0.025 mg/mL against *S. aureus* bacteria were measured. The MIC value was determined to be 0.0125 mg/mL and the MBC value to be 0.025 mg/mL against *E. coli* bacteria.

It was also reported that there is a relation between the antibacterial properties and the molecular weight of PEI [57,58]. The MIC values for PEI_600_ and PEI_1800_ against *Escherichia coli* were reported as 0.5 and 0.25 mg/mL, whereas the MIC values for PEI_600_ and PEI_1800_ against *Staphylococcus aureus* were reported as 0.016 and 0.032 mg/mL, respectively [57]. Also, Wiegand et al. reported the MIC values for PEI_800_ and PEI_5000_ against *Escherichia coli* as 0.180 and 0.122 mg/mL, respectively, whereas these values were reported as 0.150 and 0.08 mg/mL for *Staphylococcus aureus*, respectively [58]. The results obtained for the antibacterial properties of PEI with different molecular weights are in agreement with those of the literature.

In Table 4, antimicrobial properties of PEIs with different MWs after the first and second sulfobetainization reactions against different microorganisms are given.

As the antibacterial properties of b^1^-PEI and b^2^-PEI were compared, b^2^-PEIs were found to be have stronger antimicrobial effects against all the studied microorganisms. For example, the MIC value of b^1^-PEI_600_ for *C. albicans* is 200 mg/mL, while the MIC value of b^2^-PEI_600_ is 25 mg/mL. This could be due to the difference in the pH of the solutions. For example, the pH values of PEIs that were sulfobetainized a second time varied between 2.6 and 3.0. The pH values of PEIs that were sulfobetainized once varied between 5.6 and 6.3 pH. The reason why b^2^ -PEI had greater antimicrobial effects could be its pH values that are in about philological pH range. It was also reported that sulfobetaine PEI not only possessed potent antibacterial effects against bacteria such as *K. pneumoniae* and *B. subtilis* but also possessed antifungal effects against the fungus *Mucor* spp. and antiviral effects against SARS-CoV-2 virus [12]. In another study, sulfobetaine PEI has antibacterial activity, with an MIC value of 200 µg/mL and an MBC value of 400 µg/mL against *E. coli* reported [59].

Following the sulfobetainization reactions, the protonation of betainized PEI with three different acids was used to protonate the amine groups that had not been converted into zwitterions. Upon completion of the protonation with citric acid (CA), boric acid (BA), and hydrochloric acid (HCL), the resulting counterions—citrate, borate, and chloride—were associated with the positively charged amine groups. The subsequent investigation focused on evaluating the impact of these counterions on the antibacterial properties of the modified PEIs. The effects of protonation of b-PEI with different acids on antibacterial properties are given in Table 5. As seen in Table 5, protonation of second-betainization PEIs with acids improves the antimicrobial properties over the first-betainization PEIs. For example, as b^1^-PEI was protonated with HCl, the MBC value was 5 for all three microorganisms, while when b^2^-PEI was protonated with HCl, the MBC value was 25 mg/mL for all three microorganisms. It was determined that HCI is the most effective protonating agent, followed by CA. This may be due to the strong acidity of HCI.

To eliminate the effect of b-PEI due to pH, solutions of b^1^-PEI and b^2^-PEI were prepared in a BSS medium, and their pH was brought to 7 with the addition of 0.02 M NaOH. The antimicrobial properties of these solutions were examined for three different microorganisms, and the data are given in Table 6.

Upon examination of the results in Table 6, the concentration required to determine the antimicrobial behavior increases when the pH is adjusted to 7. The MBC values for the first sulfobetainization and second sulfobetainized PEI against *S. aureus* and *E. coli* bacteria are 200 mg/mL

### 3.3. Cytotoxicity of PEIs and Their Corresponding Sulfobetainized Forms

**Figure 5:**
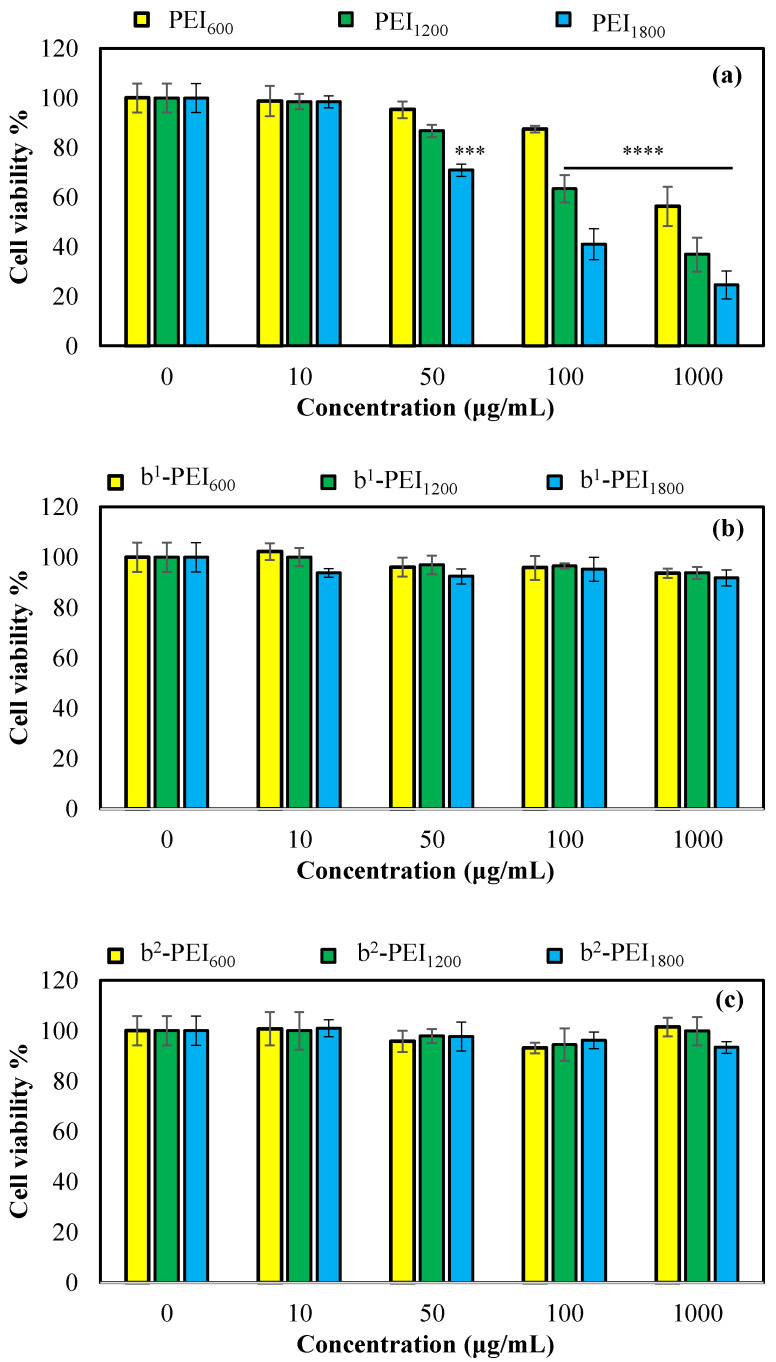
Cell viability of fibroblast cells exposed to (**a**) PEI_600_, PEI_1200_, and PEI_1800_; (**b**) b^1^-PEI_600_, b^1^-PEI_1200_, and b^1^-PEI_1800_; and (**c**) b^2^-PEI_600_, b^2^-PEI_1200_, and b^2^-PEI_1800_ for 24 h incubation time. The *p*-values as *** *p* < 0.001 and **** *p* < 0.0001 vs. control were given as statistically significant.

PEI-based materials could be used as antipathogenic material; however, their toxicity needs to be checked in advance of their use as human interaction materials and related applications. According to the ISO 10993-5 standard [60], the toxicity levels of the materials were reported to be 0, 1, 2, 3, and 4 for ≥100%, 99–75%, 74–50%, 49–25%, and 44–1% cell viability, respectively. The cytotoxicity of PEIs of different molecular weights—namely, PEI_600_, PEI_1200_, PEI_1800_—and their first and second betainized forms were tested on L929 fibroblast cells via 24 h incubation times. As seen in Figure 5a, PEI_600_ was non-toxic at 100 µg/mL concentration, with 87 ± 1% cell viability; whereas PEI_1200_ and PEI_1800_ could be safe up to 50 µg/mL concentration, with 87 ± 2% and 71 ± 2% cell viability values. Level 2 toxicity was observed for PEI_600_, with 56 ± 7% viability at 1000 µg/mL concentration; whereas PEI_1200_ and PEI_1800_ showed level 3 toxicity, i.e., below 37% cell viability values at the same concentration. Previous studies reported that the toxicity of the PEI cause from membrane destruction and necrosis effects [61], resulting from its cationic nature [62].

Therefore, the low molecular weight of the PEI represents low membrane interaction because of its low cationic charges, and it could therefore be safer than the higher-MW PEIs. Our results indicate that the toxicity level of branched PEI was slightly increased through the increase in the molecular weight. The antipathogenic activity of branched PEI at Mn 60,000 was investigated by our previous study, and its toxicity level was found to be level 4, even at a 10 µg/mL concentration. This limitation could be overcome via the sulfobetainization of more cationic PEI sources using 1,3-propane sultone [12]. Similarly, the toxicity of PEI_600_, PEI_1200_, and PEI_1800_ was improved through modification of the amine groups by in combination with SO_3_^−^ groups. As illustrated in Figure 5b,c, all PEI sources after the first and second betainization show much higher biocompatibility or biosafety, with 0–1 toxicity, up to a 1000 µg/mL concentration. Therefore, b^1^-PEI and b^2^-PEI, regardless of their MW, can be safely used in biomedical applications as they do not pose serious cell cytotoxicity.

## 4. Conclusions

The sulfobetainized PEIs with different MWs—e.g., 600, 1200, and 1800 g/mol—were successfully prepared by a simple room temperature reaction of 1,3-propane sultone with the corresponding PEIs. Moreover, the sulfobetainization reactions were carried out twice to increase the zwitterionic nature of repeating units on PEI. The increase in zwitterionic repeating units on the PEI chains was confirmed by both FT-IR and elemental analysis results e.g., with the development of the new -SO_3_ groups’ band intensity and the presence of the S element in the second betainization reaction, respectively. Moreover, it was observed that the second sulfobetainization reaction increased the thermal stability of PEI chains. Although there is a reduction in the antibacterial potency of PEI upon betainization, the toxicity of bare PEI is improved significantly by their first and second betainized forms. For example, all the sulfobetaine-modified PEIs exhibited higher safety or no toxicity, even at concentrations up to 1000 µg/mL. PEI is converted to biocompatible materials upon first and second sulfobetainization, while preserving most of the antimicrobial propensity. Sulfobetainized PEIs are also protonated with boric acid, citric acid, and hydrochloric acid to improve their antimicrobial and antiseptic properties. Therefore, betainized forms of PEI can be used as an antipathogenic spray solution against various pathogens, such as bacteria, fungus, and viruses.

## Figures and Tables

**Table 1:** Elemental compositions of PEI, b^1^-PEI, and b^2^-PEI were prepared from various molecular weights of PEI (i.e., 600, 1200, and 1800 g/mol).

Materials	wt% of C	wt% of H	wt% of N	wt% of O	wt% of S
PEI_600_	55.3	11.8	32.9	-	-
b^1^-PEI_600_	49.2	10.7	19.9	12.1	8.1
b^2^-PEI_600_	44.1	8.5	16.7	18.4	12.3
PEI_1200_	55.8	11.6	32.6	-	-
b^1^-PEI_1200_	50.3	10.9	21.8	10.2	6.8
b^2^-PEI_1200_	46.2	9.1	18.7	15.6	10.4
PEI_1800_	55.6	11.7	32.7	-	-
b^1^-PEI_1800_	52.1	11.1	23.8	7.8	5.2
b^2^-PEI_1800_	48.6	10.3	22.1	11.4	7.6

**Table 2:** Zeta potential values of PEI, b^1^-PEI, and b^2^-PEI and pH values of PEI, b^1^-PEI, and b^2^-PEI in 1 mM KNO_3_ solution.

Materials	Zeta Potential (mV)	pH
PEI_600_	−5.5 ± 3.9	10.3
b^1^-PEI_600_	6.4 ± 3.2	6.3
b^2^-PEI_600_	10.7 ± 9.9	3.0
PEI_1200_	−7.5 ± 1.0	10.6
b^1^-PEI_1200_	5.7 ± 2.4	2.6
b^2^-PEI_1200_	13.0 ± 4.1	6.2
PEI_1800_	−7.8 ± 2.5	10.7
b^1^-PEI_1800_	10.2 ± 2.9	5.6
b^2^-PEI_1800_	12.8 ± 3.1	2.7

**Table 3:** MIC and MBC values of PEI_600_, PEI_1200_, and PEI_1800_ in BSS solution against *Escherichia coli*, *Staphylococcus aureus*, and *Candida albicans*.

Materials	*Escherichia coli* (ATCC 8739)		*Staphylococcus aureus* (ATCC 6538)		*Candida albicans* (ATCC 10231)	
	MIC (mg/mL)	MBC (mg/mL)	MIC (mg/mL)	MBC (mg/mL)	MIC (mg/mL)	MBC (mg/mL)
PEI_600_	0.5	2	0.25	1	0.5	2
PEI_1200_	0.5	2	1	2	1	2
PEI_1800_	0.5	2	1	2	1	2

**Table 4:** MIC and MBC values of PEIs of different molecular wights with single or double betainization (b^1^-PEI and b^2^-PEI) in BSS solution against *Escherichia coli*, *Staphylococcus aureus*, and *Candida albicans*.

Solution	*E. coli* (ATCC 8739)		*S. aureus* (ATCC 6538)		*C. albicans* (ATCC 10231)	
	MIC (mg/mL)	MBC (mg/mL)	MIC (mg/mL)	MBC (mg/mL)	MIC (mg/mL)	MBC (mg/mL)
b^1^-PEI_600_	100	ND	100	ND	200	ND
b^2^-PEI_600_	12.5	100	25	200	12.5	25
b^1^-PEI_1200_	200	ND	200	ND	200	ND
b^2^-PEI_1200_	12.5	100	25	50	12.5	25
b^1^-PEI_1800_	200	ND	20	ND	200	ND
b^2^-PEI_1800_	50	50	100	200	25	50

Amphotericin B and ciprofloxacin were used as positive controls.

**Table 5:** MIC and MBC values of PEI_1800_ upon first and second betainization (b^1^-PEI and b^2^-PEI) with boric acid (BA), citric acid (CA), and hydrochloric acid (HCl) in a balanced salt solution (BSS) against *E. coli*, *S. aureus*, and *C. albicans*.

Microorganism	MIC (mg/mL)			MBC (mg/mL)		
	BA- b^1^-PEI_1800_	CA- b^1^-PEI_1800_	HCI- b^1^-PEI_1800_	BA- b^1^-PEI_1800_	CA- b^1^-PEI_1800_	HCI- b^1^-PEI_1800_
*C. albicans*	ND	50	50	ND	100	50
*S. aureus*	100	50	50	ND	ND	100
*E. coli*	100	100	50	ND	ND	100
Microorganism	MIC (mg/mL)			MBC (mg/mL)		
	BA- b^2^-PEI_1800_	CA- b^2^-PEI_1800_	HCI- b^2^-PEI_1800_	BA- b^2^-PEI_1800_	CA- b^2^-PEI_1800_	HCI- b^2^-PEI_1800_
*C. albicans*	25	25	25	25	25	25
*S. aureus*	25	50	25	50	50	50
*E. coli*	25	50	25	ND	ND	50

**Table 6:** MIC and MBC values of ^1^b-PEI_1800_ and ^2^b-PEI_1800_ in a BSS solution at pH 7.

Microorganism	MIC (mg/mL)	MBC (mg/mL)	MIC (mg/mL)	MBC (mg/mL)
	b^1^-PEI_1800_	b^1^-PEI_1800_	b^2^-PEI_1800_	b^2^-PEI_1800_
*C. albicans*	100	ND	200	ND
*S. aureus*	50	200	200	200
*E. coli*	50	200	200	200

Amphotericin B and ciprofloxacin were used as positive controls.

## Data Availability

All the data generated in this study are used within the study.

## Supplementary Materials

The following supporting information can be downloaded at www.mdpi.com/xxx/s1:; Table S1. Theoretically calculated elemental compositions of PEI, b1-PEI, and b2-PEI prepared from various molecular weights of PEI such as 600, 1200, and 1800 g/mol.
