# Supplementary material for: Toxicity Evaluation of Sulfobetainized Branched Polyethyleneimine via Antibacterial and Biocompatibility Assays
Source: Toxics. 2025 Feb 14;13(2):136. doi: 10.3390/toxics13020136 (PMC11860753; doi:10.3390/toxics13020136)
Supplement: Supplementary file 1 [file toxics-13-00136-s001.zip › toxics-3425561-supplementary.pdf]

Toxicity evaluation of sulfobetainized branched polyethyleneimine via antibacterial and biocompatibility assays

Mehtap Sahiner[2,3], Selin S. Suner[3], Sahin Demirci[4], Ramesh S. Ayyala[5], Nurettin Sahiner[3,5,6,*]

1 Department of Bioengineering, Faculty of Engineering, Canakkale Onsekiz Mart University, Terzioglu Campus, 17100 Canakkale, Turkey; sahinerm78@gmail.com
2 Department of Chemical, Biological, and Materials Engineering, University of South Florida, Tampa, FL 33620, USA
3 Department of Chemistry, Faculty of Sciences, Canakkale Onsekiz Mart University, Terzioglu Campus, 17100 Canakkale, Turkey; sagbasselin@gmail.com
4 Department of Food Engineering, Faculty of Engineering, Istanbul Aydin University, Florya Halit Aydin Campus, 34153 Istanbul, Turkey; sahindemirci@gmail.com
5 Department of Ophthalmology, Morsani College of Medicine, University of South Florida, 12901 Bruce B. Downs Blvd, MDC21, Tampa, FL 33612, USA; rayyala@usf.edu
6 Department of Bioengineering, U.A. Whitaker College of Engineering, Florida Gulf Coast University, 10501 FGCU Boulevard South, Fort Myers, FL 33965, USA
* Correspondence: nsahiner@fgcu.edu or sahiner71@gmail.com

Table S1. Theoretically calculated elemental compositions of PEI, b1-PEI, and b2-PEI prepared from various molecular weights of PEI such as 600, 1200, and 1800 g/mol.

Materials	wt% of C	wt% of H	wt% of N	wt% of O	wt% of S
PEI_{600}	55.8	11.6	32.6	-	-
b^1-PEI_{600}	44.9	8.8	19.1	16.3	10.9
b^2-PEI_{600}	40.4	7.6	13.5	23.1	15.4
PEI_{1200}	55.8	11.6	32.6	-	-
b^1-PEI_{1200}	44.9	8.8	19.1	16.3	10.9
b^2-PEI_{1200}	40.4	7.6	13.5	23.1	15.4
PEI_{1800}	55.8	11.6	32.6	-	-
b^1-PEI_{1800}	44.9	8.8	19.1	16.3	10.9
b^2-PEI_{1800}	40.4	7.6	13.5	23.1	15.4
